# Liquid biopsies: the future of cancer early detection

**DOI:** 10.1186/s12967-023-03960-8

**Published:** 2023-02-11

**Authors:** Siobhan Connal, James M. Cameron, Alexandra Sala, Paul M. Brennan, David S. Palmer, Joshua D. Palmer, Haley Perlow, Matthew J. Baker

**Affiliations:** 1 Dxcover Ltd., Royal College Building, 204 George Street, Glasgow, G1 1XW UK; 2 Department of Pure and Applied Chemistry, Thomas Graham Building, University of Strathclyde, 295 Cathedral Street, Glasgow, G11XL UK; 3 Translational Neurosurgery, Centre for Clinical Brain Sciences, 49 Little France Crescent, University of Edinburgh, Edinburgh, EH16 4BS UK; 4 Department of Radiation Oncology, The Ohio State University Wexner Medical Center, Columbus, OH 43210 USA; 5 School of Medicine, Faculty of Clinical and Biomedical Sciences, University of Central Lancashire, Preston, PR1 2HE UK

**Keywords:** Liquid biopsy, Multi-cancer, Early detection, Cancer, Diagnostics

## Abstract

Cancer is a worldwide pandemic. The burden it imposes grows steadily on a global scale causing emotional, physical, and financial strains on individuals, families, and health care systems. Despite being the second leading cause of death worldwide, many cancers do not have screening programs and many people with a high risk of developing cancer fail to follow the advised medical screening regime due to the nature of the available screening tests and other challenges with compliance. Moreover, many liquid biopsy strategies being developed for early detection of cancer lack the sensitivity required to detect early-stage cancers. Early detection is key for improved quality of life, survival, and to reduce the financial burden of cancer treatments which are greater at later stage detection. This review examines the current liquid biopsy market, focusing in particular on the strengths and drawbacks of techniques in achieving early cancer detection. We explore the clinical utility of liquid biopsy technologies for the earlier detection of solid cancers, with a focus on how a combination of various spectroscopic and -omic methodologies may pave the way for more efficient cancer diagnostics.

## Background

Cancer is one of the leading causes of death worldwide, accounting for almost 10 million deaths in 2020, with around 19.3 million new cases reported each year [1, 2]. Cancer accounts for nearly one in every six deaths [3]. Identification of aggressive tumors at an earlier stage can enable more effective treatment [4]. This would not only improve the quality of cancer patients’ lives but also improve survival rates of many cancers. At later disease stages, surgery is markedly less effective, radiotherapy more likely indicated, and chemo-therapeutic drugs are often more toxic. Diagnostic delays result in a poorer patient outcome, and the medical expenses associated with medication, home and clinical medical visits, and in-hospital care increase significantly with cancer stage [5, 6].

The overall age standardised incident rates of cancer in low- and middle-income countries (LMICs) are reported as lower than incident rates in high-income countries (HICs); however, the total cancer related mortality is considerably higher in LMICs, particularly for people younger than 65 years of age [7, 8]. The burden of cancer in LMICs adds stress to an already weak health care and poor economic infrastructures, moreover, this burden is not captured in an accurate way due to the lack of reliable cancer registries and reporting systems [7, 8]. Cancer survival rates have been continually improving within HICs, thanks to earlier diagnosis and more advanced treatments [9]. As a result, cancer- control strategies developed and effective in HICs are often not applicable or useful in LMICs due to differences in disease characteristics and profound deficiency in health system capabilities [8]. Although, this is also due to the imbalance in the resources allocated for cancer research in HICs v LMICs [8].

The analysis of cancer related signals using biological fluids—a liquid biopsy—has generated great interest in the past decade. Liquid biopsies can identify a wide range of biomolecular features and have the potential to give an indication of disease status. The liquid biopsy market is expected to increase at rate of ~ 16% between 2020 and 2030 [10]. However, many existing liquid biopsies with a focus on early cancer detection lack the sensitivity required for reliable detection of early stage cancers [11]. For example, tumor derived genetic biomarkers are not always shed into the blood stream in early stages, and even when they are shed in to the bloodstream, they exist at very low concentrations [12, 13]. Cancer protein biomarkers such as prostate specific antigen (PSA) and carcinoma antigen-125 (CA-125) are often not elevated in cancer patients, even in those with advanced cancer [14]. Furthermore, they lack specificity as these markers can also be elevated in patients without cancer [15–17]. For more effective early cancer detection, technologies require consideration of non-tumor derived information as well as signals directly from the tumor [18]. This review discusses the liquid biopsy techniques currently under investigation and their potential for early-stage detection of solid carcinomas.

### Impact of earlier cancer detection

**Fig. 1:**
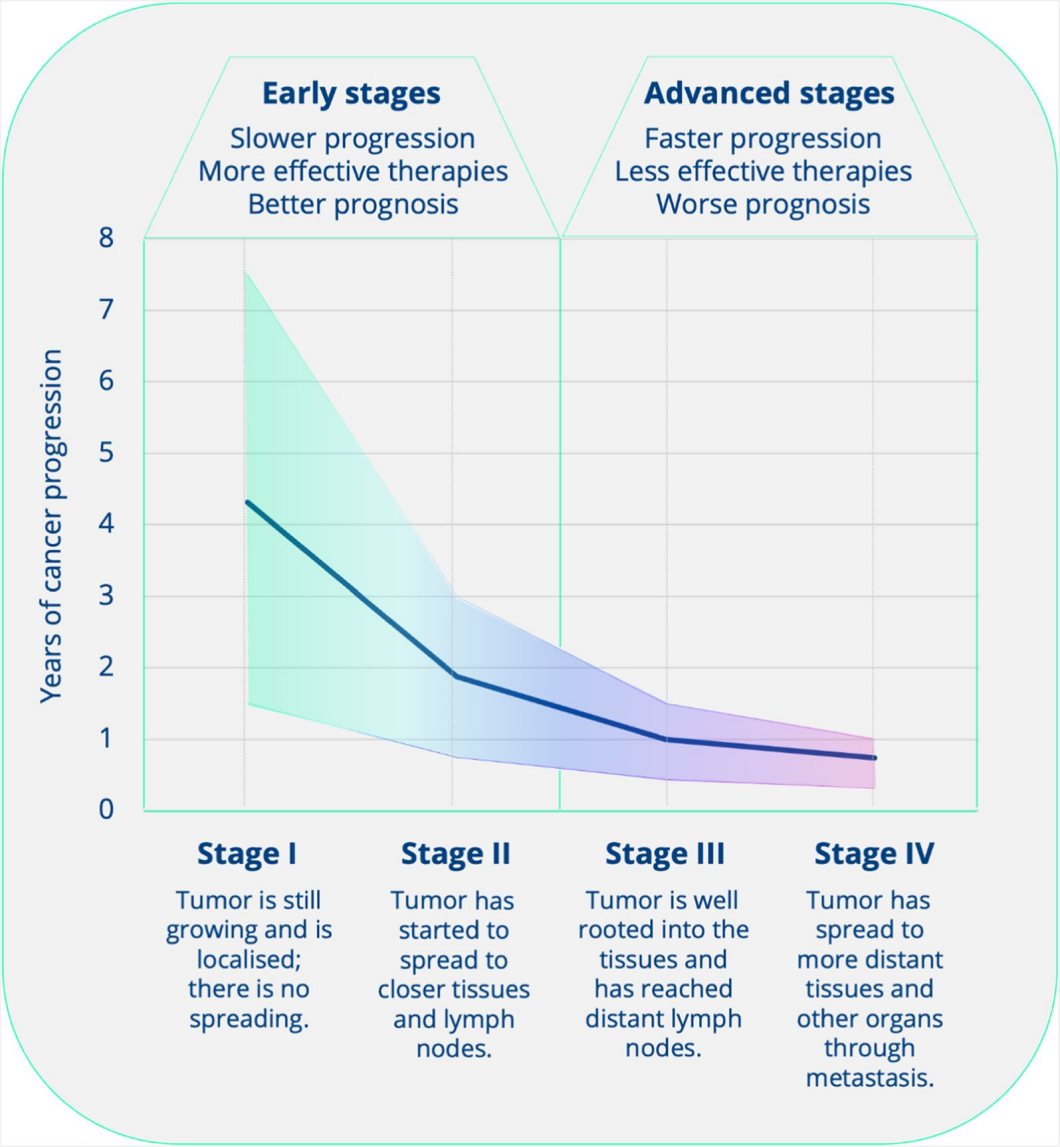
Cancer progression with time, data adapted from [19]

Most cancers can be classified according to the stage of disease, a measure of how widely it has spread in the primary organ and beyond: stage 0 (i.e., in situ), I, II, III and IV. Localized disease refers to cancer that is contained where it started with no sign of spreading (Stages 0-I-II). Regional disease represents spread of the cancer to nearby, organs, tissue, or lymph nodes (Stages II-III). Distant disease is often referred to as metastatic cancer and relates to cancer which has spread to other areas of the bodies (Stage IV). Higher stage cancers are more difficult to effectively treat. As the tumor stage progresses from I to IV, the growth rate increases and the time period to the next stage decreases (Fig. 1) [19].

**Fig. 2:**
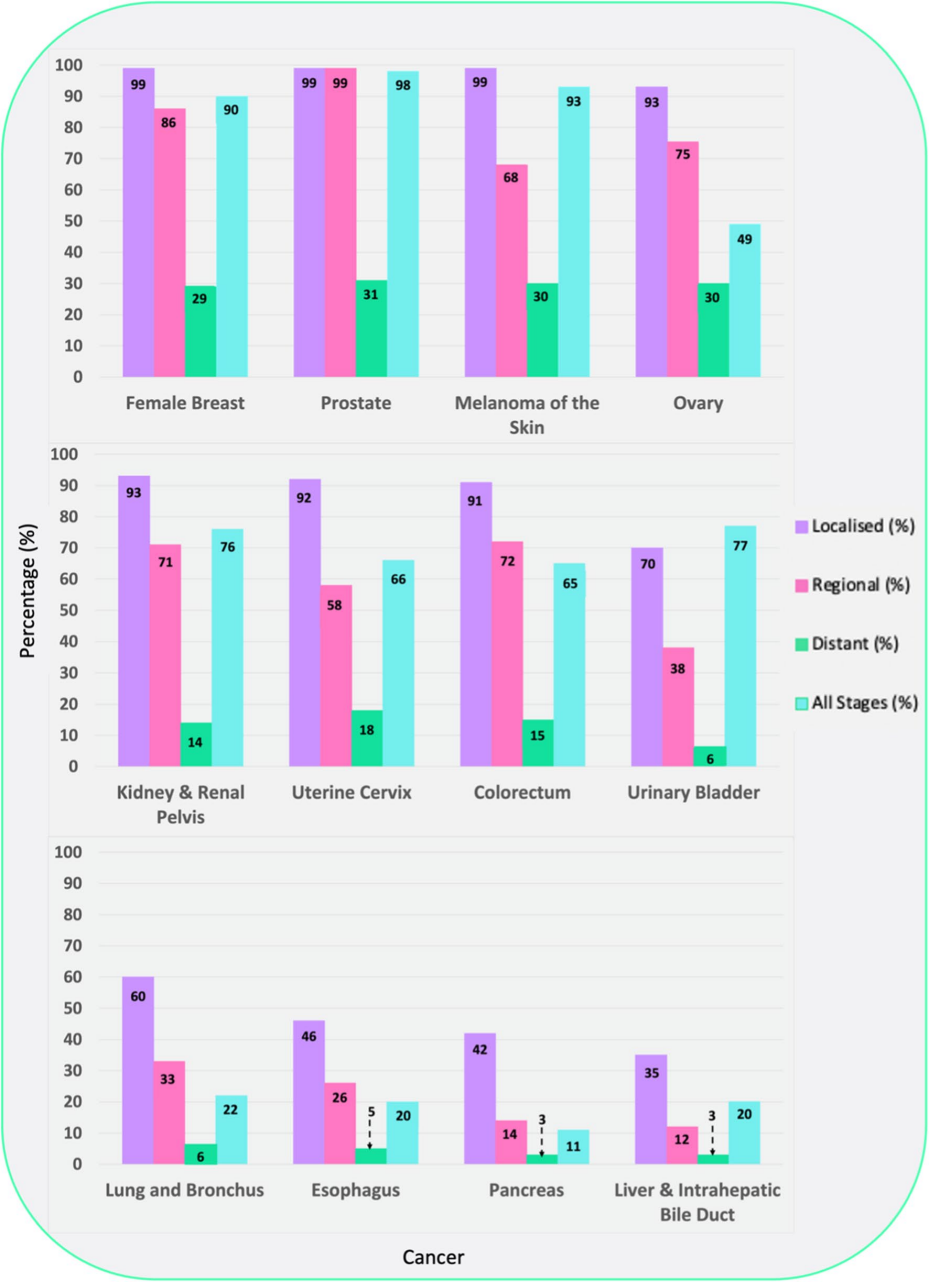
Five-year relative survival for selected cancers by stage at diagnosis, United States 2011 to 2017. Adapted from [21]

Cancer metastasis is the spread of cancerous cells to organs and tissues beyond the primary tumor site leading to the possible formation of secondary tumors. Metastatic lesions are the leading cause of death in cancer patients, accounting for 90% of all cancer-related deaths [20] Fig. 2 highlights the five-year relative survival for selected cancers by stage at diagnosis, demonstrating the impact of late-stage cancer diagnosis on survival [21]. Other factors such as tumor size, location, type, and number of metastatic lesions, also impact on survival. The general trend shows a decreasing survival rate with increasing cancer stage.

**Fig. 3:**
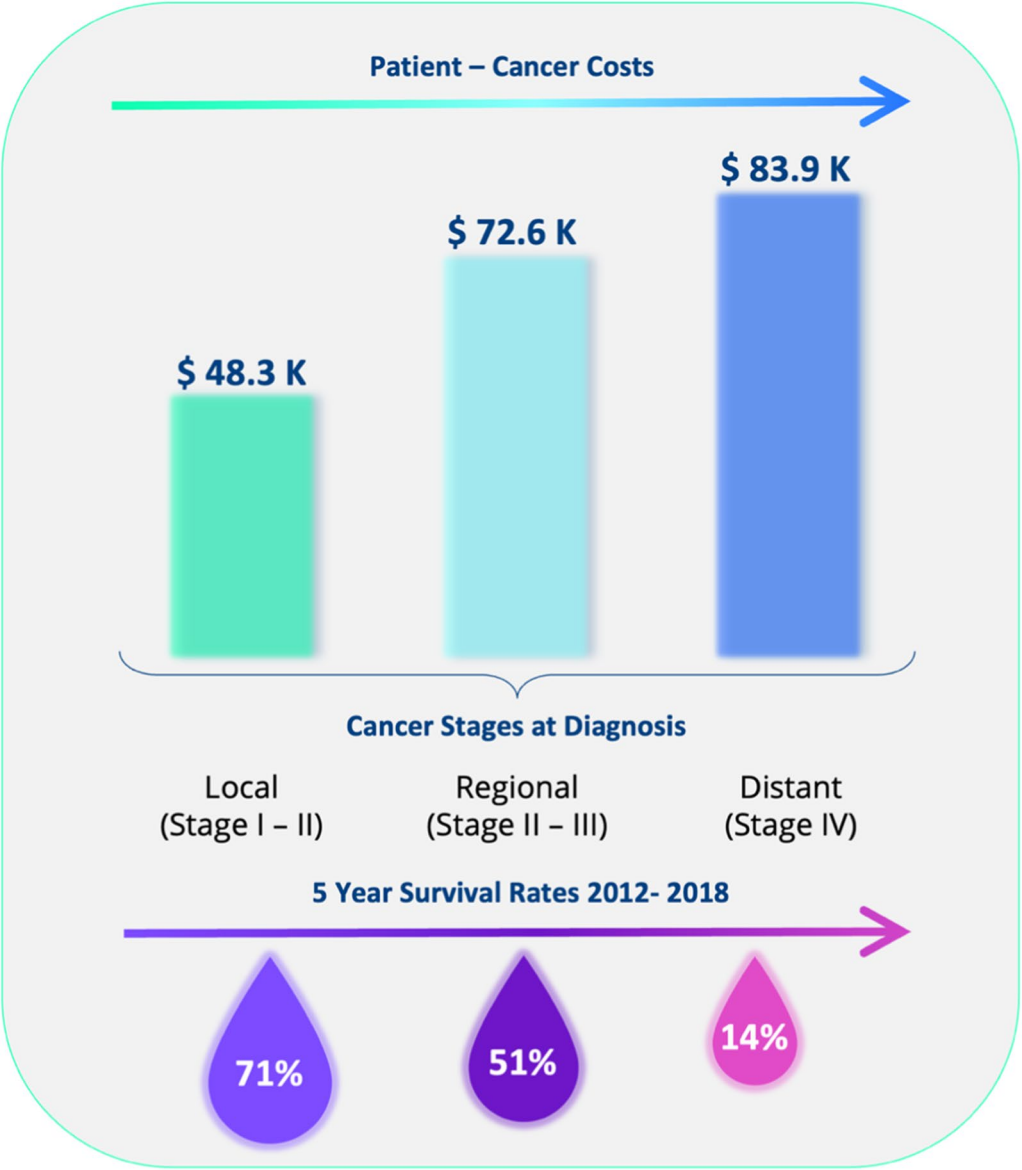
Patient cancer cost associated with the first 12 months averaged over 11 cancer types (bladder, breast, colorectal, esophagus, kidney, liver, lung, ovary, pancreas, prostate and stomach), data adapted from [22]. Survival rates, averaged from SEER 5 -Year Survival Rates 2012–2018 across 11 cancer types (bladder, female breast, colorectal, esophagus, kidney, liver and intrahepatic bile duct, lung and bronchus, ovary, pancreas, prostate and stomach) data from both sexes unless stated otherwise, calculated from [28]

The cost of treating patients diagnosed with stage III/ IV cancer is dramatically increased in comparison stage I/II cancer (Fig. 3) [22, 23]. Differences in costs between higher and lower stage disease reflects shorter hospital stays, reduced outpatients visits and lower numbers of emergency admissions associated with early stage cancer [24]. Most patients (~ 70%) diagnosed with Stage I cancer undergo surgery as part of their treatment where possible—surgery has shown to provide the best chance of curing the cancer and with fewer side effects in comparison to chemotherapy and radiotherapy [25]. Earlier diagnosis of cancer will save lives and significantly reduce treatment costs [26]. Yet current clinical tests lack sensitivity and specificity in early-stage cancers [27]. In fact, many cancers are asymptomatic in the early stages [12].

### Screening, triage and diagnosis

Triage is the process of stratifying symptomatic patients in terms of clinical urgency [29, 30]. A triage test can support clinicians to determine which patients are most likely to have a disease and should be fast tracked for diagnostic tests [29]. The utility of a triage test depends on factors, such as the prevalence of the disease, the target population, the performance characteristics of the test itself, and the availability of resources for downstream investigations [29].

**Fig. 4:**
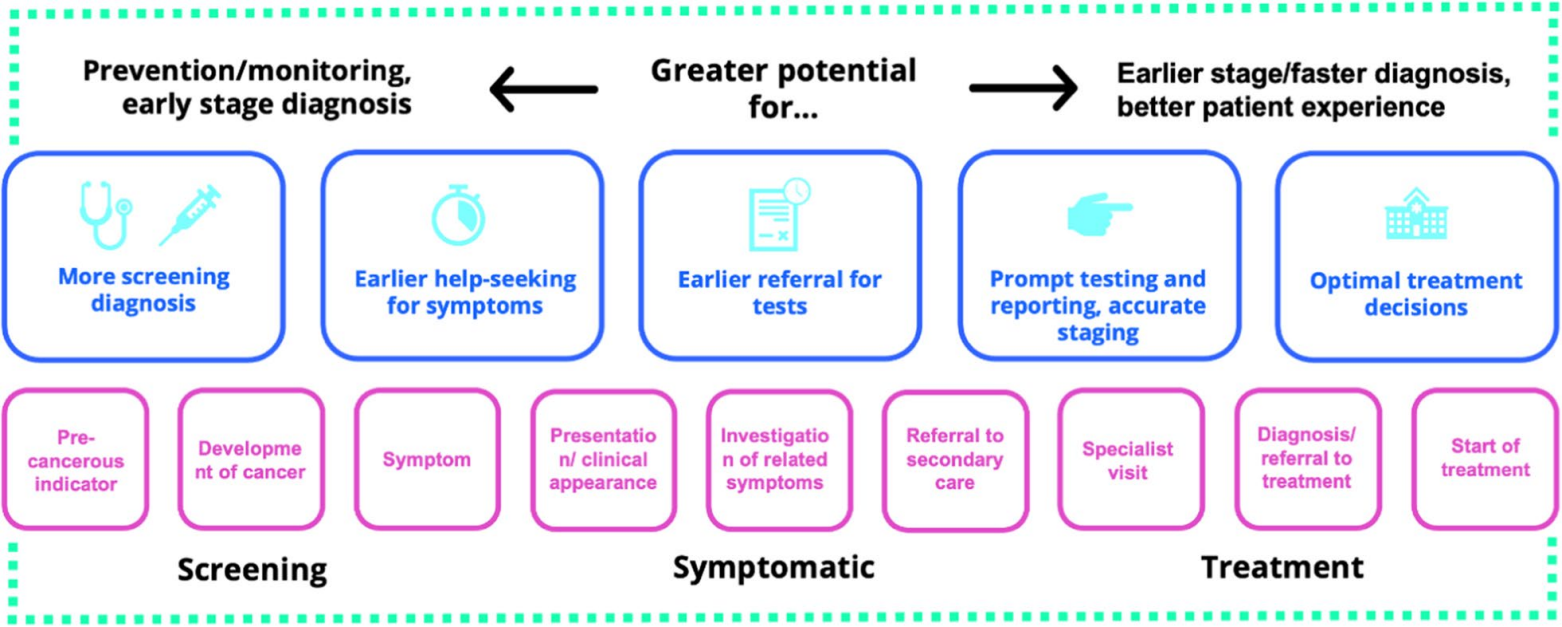
Acting across the diagnostic pathway, adapted from [35]

A cancer screening test is performed in asymptomatic patients and has normally one of two aims; to reduce the mortality and morbidity in a population through early detection and early treatment of cancer (e.g., breast screening) or to reduce the incidence of a cancer by identifying and treating its precursors (e.g., cervical screening) [31]. In the UK screening tests are available for breast, cervical and bowel cancer, and in the US also prostate and lung cancer [32, 33]. Screening for individual cancers is expensive, and in the future, it may be more efficient to use a multi-cancer test (Fig. 4) that can detect a range of cancers from a single test. Such a test would also be valuable in the triage of patients presenting with non -specific symptoms, where the suspicion of cancer is low. A low-cost blood test could help doctors triage patients with non-specific symptoms and a low suspicion of cancer for rapid early investigation.

The specificity of screening and triage tests for cancer is critical. Identification of pre-cancer or early-stage cancer allows timely treatment, but tests with low specificity are associated with high incidences of false positive results, leading patients to be subjected to more, often invasive tests. Where a slow-growing tumor is identified, which is unlikely to have been problematic for the patient, harm may instead result from further investigations [34]. Improving the specificity of a test usually results in a lowering of the test sensitivity, which would mean more cancers are not detected, so-called false negatives test results. The trade-off between sensitivity and specificity acceptable to patients and medical professionals needs to be determined for each test and depends in part on the consequences of the false result.

The diagnosis of cancer in screen positive or symptomatic patients requires imaging tests and often tissue biopsy. Imaging techniques such as a computed tomography (CT) scan and/or a magnetic resonance imaging (MRI) scan are relatively expensive, costing the UK’s NHS around $145.69* (£120) per CT and approximately $262.24* (£216) per MRI $242.81–364.22* (£200–300) [36] (*based on an exchange rate of $1.21to £1) and approximately $3275 (CT) and $1325 (MRI) [37, 38] in the US. These costs affect the threshold for referring patients for imaging. Both screening and symptom-based triage tests have a low specificity, so most imaging investigations are then true negatives.

Tissue biopsies are regarded as the “gold standard” for tumor profiling in cancer diagnostics [39] and are required in majority of cases to determine the specific type of cancer [40]. A biopsy can be obtained in several ways depending on the tumor location and surgical treatment plan, for example through endoscopy or needle biopsy [40]. If the sample obtained is too small, this can lead to misdiagnosis. In excisional biopsy, an entire area of abnormal cells is removed, whereas in an incisional biopsy just a part of the abnormal area is removed [40]. Open surgical biopsies enable more precise resections, but carry increased risk of complications, such as infections or bleeding. Moreover, one of the main issues related to tissue biopsies is the inability to capture tumor heterogeneity and its clonal evolution, which can be obtained using liquid biopsy approaches.

A liquid biopsy test could enhance the screening and triage pathways and increase the proportion of patients referred for onward investigation who have an abnormality. This increased efficiency in the diagnostic process would reduce the delay to diagnosis, as well as costs [41]. An effective liquid biopsy triage test needs to be low cost, so it can be applied in the large population with non-specific symptoms and will ultimately reduce the number of unnecessary diagnostic procedures performed, reducing overdiagnosis, overtreatment, patient anxiety, as well as costs. A liquid biopsy triage test that can detect multiple cancers would be desirable in patients with non-specific symptoms.

### Liquid biopsies

Liquid biopsy is an all-encompassing term used to describe the testing of bodily fluids including, blood, urine, cerebrospinal fluid, and saliva. Definitions of liquid biopsy within the cancer diagnostics field tend to focus on tests that target specific biomarkers. The National Cancer Institute states that a liquid biopsy is; “*A test done on a sample of blood to look for cancer cells from a tumor that are circulating in the blood or for pieces of DNA from tumor cells that are in the blood”* [42]. Many publications also define liquid biopsies with a similar narrow viewpoint:

*“… a test to search for cancer cells or pieces of DNA from tumor cells in a blood sample, liquid biopsies can serve a variety of purposes” *[43].

*“…liquid biopsy—the analysis of tumors using biomarkers circulating in fluids such as the blood…” *[44].

**Fig. 5:**
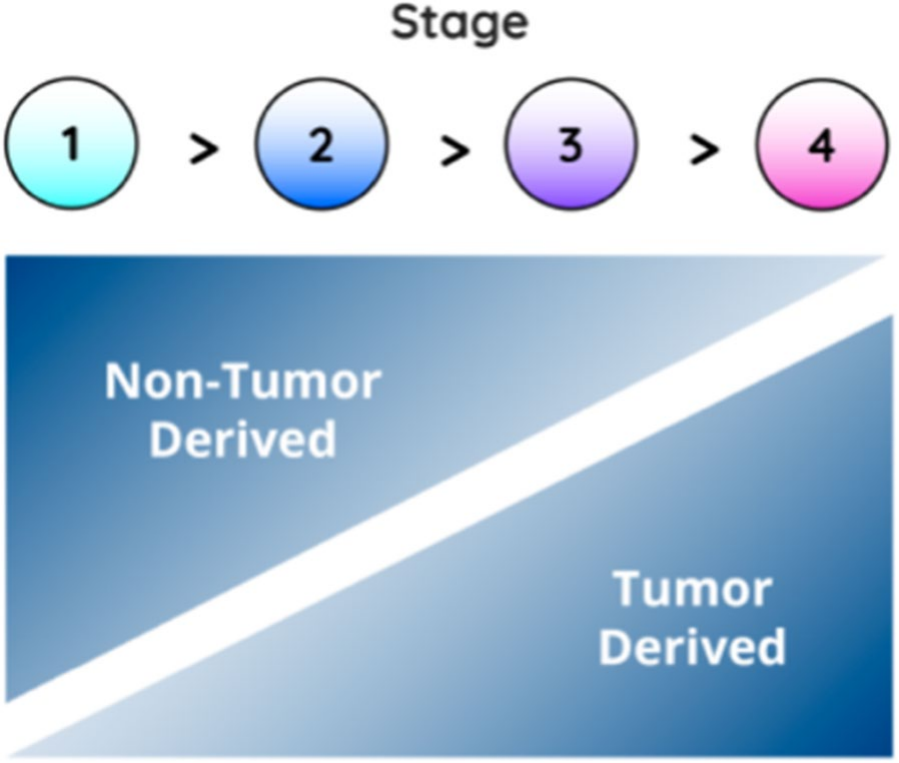
Tumor and non-tumor derived information prevalence varying with stage, adapted from [46]

Although, cancer is a systemic disease and not all biomarkers relate directly to the cancer cell. As a cancerous lesion evolves and grows, the biological signals change. In the early stages, non-tumor derived sources–such as the immune response—dominate [45, 46]. The immune response plays an important part in the regulation of initiation and progression of tumors [18]. The small size of early tumors means that the level of tumor-related biomarkers shed into circulation will be very low, making reliable and accurate detection a significant challenge [18]. By contrast, systemic, non-tumor derived markers are likely to be more prevalent. A combination of both tumor and non-tumor derived signals, in a pan-omics approach could lead to the successful early detection of cancer (Fig. 5) [45, 46].

There are several benefits of liquid biopsy over conventional surgical tissue biopsy. Liquid biopsies have lower procedural costs [47, 48], are easily repeatable, and are more reliable [44]. This therefore could make liquid biopsies more suitable and accessible for use in low- and middle-income countries. Surgical tissue biopsies are not attainable for some cancers due to the high risk associated with the procedure. Sample heterogeneity, which can lead to misdiagnosis of surgical biopsies, is not an issue with liquid biopsy [10]. Liquid biopsies are not contaminated from the use of preservatives, whereas tissue sections are generally preserved for immunohistochemistry by processes such as fixation, embedding and freezing. Liquid biopsies provide a fresh source of reliable tumor-derived components and materials [49]. Furthermore, liquid biopsies can be carried out rapidly, provide genomic, proteomic and metabolomic information, and are less invasive than tissue biopsies [4, 48, 49].

Currently liquid biopsies are not considered a standard method for the diagnosis and conformation of diseases such as cancer [50]. Instead, they are predominantly used as a complementary test to tissue biopsy. This is related to liquid biopsies in comparison to tissue biopsies being generally less sensitive and specific, which can lead to an increase in the occurrence of false positives and false negatives [50, 51]. In turn this can cause a delay to a patient receiving a correct diagnosis and the appropriate treatment. Liquid biopsies are also associated with elevated economic costs [50]. Moreover, current liquid biopsies lack the required accuracy in predicting tumor origin in patients who test positive [11, 52]. This inability can pose challenges for clinical follow-up, yet there is still some promise and with further development the sensitivity may be enhanced for this application.

### Current liquid biopsy techniques for detection of cancer

#### Isolation and sample preparation

Many genetic technologies require complex multi-step processes for sample preparation—these processes can be both time consuming and costly. For example, deoxyribonucleic acid (DNA) assays typically go through a five-stage process for DNA extraction [53]. Firstly, the cellular structure is disrupted to create a lysate, the soluble DNA is then separated from cell debris and any other insoluble material. The DNA of interest is then bound to a purification matrix, where after proteins and any other contaminants are washed away the DNA can then finally be eluted [53]. Throughout this process yield, purity and integrity are essential factors as this will affect the performance of applications later in the process such as enumeration.

#### Circulating tumor cells

Circulating tumor cells (CTCs) were first described by Ashworth in 1869 [27]. CTCs are released into the blood by a tumor, and travel through the blood stream or lymphatic system to other areas of the body—having the potential to cause distant metastases [27, 47, 48]. The initial applications of liquid biopsy in the cancer field were focused on CTCs [48, 54]. CTCs have different molecular markers depending on the type of cancer [55]. However, since most cancers are of epithelial origin, there is a ‘universal’ epithelial molecular marker, EpCAM, which can be used for CTC detection. The expression of EpCAM varies with different cancer types and is mainly applied to cancers such as breast and prostate which strongly express EpCAM.

CTCs occur at a very low concentrations (< 10 CTCs per mL of blood) in circulation, even in patients with metastatic cancer [12]. Therefore, highly sensitive technologies are required to efficiently detect and isolate these cells, from the millions of other blood cells present [56, 57]. Furthermore, the utility of CTCs for use as a method for early detection of cancer is limited—since the number of CTCs present in blood samples has been seen to correlate with clinical staging, with the highest numbers generally found in patients with late-stage aggressive cancer—which can still be very low [57]. The variation of CTC markers highlights the heterogeneity of CTCs between different cancer types, also presenting variation between cancer stages and during treatment periods [55]. Since there are currently only a limited number of molecular markers available, it is difficult to define the entire CTC population.

The CellSearch system—a blood test used for the identification, isolation, and enumeration of CTCs of epithelial origin was approved by the Food and Drug Administration (FDA) for clinical use to assess the prognosis of patients with metastatic breast, colorectal and prostate cancer [58–60]. This test has a turnaround time of one week and cost approximately $900 (December 2016) [61]. Other observational studies within metastatic prostate cancer have highlighted that CTC’s can be utilized to monitor progression on systematic treatment with the potential to stop ineffective treatment earlier [27].

#### Cell-free DNA/circulating tumor DNA

Cell-free DNA (cfDNA) is the fragmented DNA found in biofluids released from cells into the circulatory system [4, 39, 62, 63]. It is released from cells mainly through apoptosis (programmed cell death), necrosis (accidental cell death) and active secretion from the tumor [47, 62]. It was first observed by Mandel and Métais in 1948 and can be found in many body fluids, such as blood (plasma and serum), urine, saliva and cerebrospinal fluid, and is present in both healthy and diseased patients [62–64]. cfDNA from healthy cells are found at low levels in plasma (~ 10–15 ng/mL); however, it has been reported that cfDNA concentration can increase upon tissue stress induced by inflammation, surgery, acute trauma [62] and exercise [39]. Since its discovery, cfDNA has become an appealing biomarker, and the analysis of cfDNA has been utilized in a range of medical technologies, such as prenatal testing, detecting immune diseases, monitoring the effectiveness of an organ transplant, and detecting the presence of cancer [63].

Fragmented tumor DNA in the blood stream is known as circulating tumor DNA (ctDNA) [4]. In people with cancer this accounts for around 1 to 2% of the overall cfDNA [65, 66]. ctDNA can be distinguished from normal cfDNA fragments through the presence of epigenetic or genetic alterations including tumor-specific methylation markers and somatic mutations [67]. ctDNA can be used as a marker for treatment selection, to estimate prognosis, as well as for identification of residual disease and/or indicating potential risk of relapse [5]. One study reported that ctDNA assays were able to detect residual disease faster than radiologic imaging by several weeks [5].

There are some limitations to cf/ctDNA strategies. The detection capability required for early-stage cancers is often beyond that of current techniques [52]. From observational studies the half-life of cfDNA in the circulatory system varies, between 1 min to 2.5 h [4, 62] and cf/ctDNA levels are generally very low, so that detection has been compared to “searching for a needle in a haystack” [13]. The release of cf/ctDNA into the blood stream is highly variable and although the concentration in plasma has been shown to correlate with both the tumor stage and size [5], it is only found in 75% of patients with metastatic disease [27]. Bettegowda et al. highlighted that the fraction of patients with detectable ctDNA (with either breast, colon, pancreas or gastroesophageal cancer) was 47%, 55%, 69% and 82% for patients with stage I, II, III and IV cancers respectively [14]. This demonstrates that there is a vastly different response associated with the cancer stage and the amount of ctDNA released into the blood stream. Moreover, It has been highlighted that in order to achieve 95% sensitivity for breast cancer screening approximately 150 to 300 mL of blood would be required per test [5].

#### Methylation markers

DNA methylation is an epigenetic mechanism involving the enzymatic transfer of a methyl group onto the carbon-5 position of cytosine to form 5-methlycytosine [68, 69]. DNA methylation occurs naturally in the body, however abnormal patterns of DNA methylation have been identified as indicators of diseases such as cancer [70]. DNA methylation changes have been reported to occur in carcinogenesis and can be found in detached tumor cells within bodily fluids and biopsies [69]. Moreover, they also have the potential to be used as a method of risk assessment for the future development of disease [69]. For most of the current technologies that detect DNA methylation markers within body fluids, the sensitivity is relatively low, with a substantially higher specificity.

Bisulfite genomic sequencing is considered the ‘gold standard’ for the detection of DNA methylation due to its ability to identify 5 – methylcytosine (5mC) at single base-pair resolution [70, 71]. It provides a qualitative, quantitative, and an efficient approach since cytosine and 5-methylcytosine react differently upon treatment with sodium bisulfite. Cytosine from single stranded DNA will be converted firstly into uracil residues via the process of deamination, which will then be recognized as thymine in subsequent PCR amplification and sequencing. However, 5mCs are untouched by this process as they are thermodynamically protected, allowing the distinction between methylated and unmethylated cytosines [70, 71]. During PCR amplification any bisulfite-converted fragments (uracil’s) are replaced with thymine’s, creating a DNA sequence which can be compared with a reference, unconverted, DNA sequence to determine the extent of the cytosine methylation [71]. However, bisulfite treatment is labor and computationally intensive, and is also susceptible to bias from incomplete bisulfite conversion. Harsh chemical and temperature conditions are required which can result in the significant loss of materials through DNA degradation, which is then harder to PCR amplify [72].

The development and introduction of cancer-specific methylation markers will allow the introduction of small panels of markers suited for certain clinical applications. In terms of screening, a panel consisting of the most common cancer -specific methylation markers (multiple methylation markers are common across multiple cancers) will allow more diagnostic information to be obtained in terms of the tissue of origin, moreover it will also be a lot more efficient than a single-assay marker would [69].

#### Extracellular vesicles

Extracellular vesicles (EVs) are small membranous particles, which can be found in the majority of bodily fluids—especially blood [47, 67, 73]. EVs are fundamental mediators of intercellular communication [48, 67], as they regulate a vast amount of both pathological and physiological processes [47, 48]. There are three main categories of EVs; exosomes, microvesicles (MVs) and apoptotic bodies, which are differentiated on their size, content, function, release pathways and biogenesis [73]. Each of the three subtypes of EV’s have different protein profiles relating to their different routes of formation. EVs carry and transport a variety of different biomolecular components, such as lipids, carbohydrates, proteins, metabolites, ribonucleic acids (RNAs) and DNA fragments [67]. Additionally, isolated EVs from the biofluids of cancer patients have been reported to contain tumor derived molecules. The molecular information carried by EVs are thought to be a molecular fingerprint of the cell of origin, thus they are being considered as a potential cancer biomarker [47, 74, 75].

EV’s have advantages over ctDNA and CTCs as a tool for liquid biopsy; they have a double-layered membrane structure which makes them less easily degradable than nucleic acids and they also maintain the original source of cellular biological information well [76]. Limitations surrounding the clinical suitability of EVs are related to the lack of standardized protocols and the variability between different isolation techniques [47, 67, 73]. Moreover, obtaining blood derived EVs with a high purity is difficult, as they can be obscured by other components in blood such as cells, lipoproteins and cfDNA [67].

EV’s can either work for or against cancer—they have the ability to promote the spread of cancer cells, creating a suitable environment for cancer metastasis, aiding its development and progression [76]. However, assisting in the occurrence and spread of cancer also reveals the existence of cancer and so EV’s have become an effective way for both diagnosing and treating the disease. EV-based blood biomarker classifiers based on EV protein profiles have been used to detect stage I and II pancreatic, ovarian and bladder cancer [77]. Moreover, the ExoDx Prostate IntelliScore is an example of a non-invasive exosome based liquid biopsy used to identify patients at risk of high-grade prostate cancer [78].

#### Proteins

Liquid biopsies based on the detection of protein biomarkers have great potential for cancer detection and monitoring of the disease progression [79]. Proteins carry out many of the cellular functions within cells, therefore proteomic data may be able to aid novel biomarker identification and clinical implementation [80]. However, current protein assays fail to reach the required diagnostic accuracy [79, 80]. Research into different methods to enhance the diagnostic accuracy and subsequently reduce the number of false positives and negatives include the use of panels or biosignatures comprising of more than one protein [47], as well as a combination of both protein and DNA biomarkers [79].

The prostate-specific antigen is an example of a protein biomarker which is currently used for the identification of prostate cancer, but there are questions over its clinical utility. Elevated PSA levels are not specific to prostate cancer; common conditions such as prostatitis and benign prostatic hyperplasia can impact the levels observed [81]. Moreover, there are several factors such as age, race, body mass index, medication as well as others which must be considered before determining what ‘elevated’ PSA levels are. A study examining 6 randomized control trials totaling 390,00 men aged between 45 and 80 highlighted that routine screening for prostate cancer had no statistically significant effect on all-cause mortality, death from prostate cancer or on the diagnosis of stage III or IV prostate cancer [82]. Although there was an increase in the probability of being diagnosed with cancer especially stage I—for approximately every 1000 men screened there was on average 20 more cases of prostate cancer diagnosed. Another study identified that up to 42% of men diagnosed with prostate cancer are individuals that would never have developed clinical symptoms in their lifetime [83]. High levels of false positive results can expose patients to unnecessary follow up appointments and procedures [84].

Cancer Antigen-125 is a tumor biomarker which over the last four decades has been utilized as the primary ovarian cancer biomarker [85]. CA-125 is found on the surface of ovarian cancer cells and is a high molecular weight glycoprotein [86]. Techniques used to detect CA-125 lack the sensitivity (> 75%) and specificity ($\ge 99.6 \%)$ required to be used in a general-population screening program for detection of ovarian cancer [85, 86]. Increased levels of serum CA-125 are found in 75–90% of advanced stage tumors, yet only in 23–50% of early stage tumors, suggesting this biomarker is not suitable for early stage detection [17, 85, 86]. CA125 is also not specific solely to ovarian cancer with elevated serum CA-125 levels also observed in menstruation, endometriosis and pregnancy, so false positive for cancer can be an issue [17].

#### Ribonucleic acid

Cell free RNA (cfRNA) are RNA fragments which are degraded and released into the bloodstream mainly by necrotic or apoptotic cells [87]. Circulating tumor RNA (ctRNA) refers to the fraction of circulating cell-free RNA derived from cancer cells. RNA in comparison to DNA is regarded as an unstable molecule with a ‘naked’ half-life in plasma of approximately only 15 s [47], this lack of stability is one of the major limitations associated with ctRNAs, and an optimal extraction method has yet to be identified.

Cell–free messenger RNA (mRNA) was first confirmed in the bloodstream of patients with cancer in 1999, leading to mRNA being identified as a potential cancer biomarker with prognostic and diagnostic value [87]. The research surrounding non-coding RNA (ncRNA) has increased particularly in small RNAs for potential use as prognostic and diagnostic disease biomarkers, due to their higher stability and abundance. MicroRNA (miRNA) has gained the most interest due to its stability, moreover in most human cancers the miRNA levels are altered, and its expression is tissue specific. MicroRNA can be detected not only in tissue samples but also in serum and urine, as well as other accessible sources using minimally invasive techniques [88]. Drokow et al*.* conducted a meta-analysis study to provide a comprehensive evaluation of the overall accuracy of miRNA detection in the diagnosis of hematological cancer. The pooled specificity was 85%, with a sensitivity of 81%, highlighting that miRNAs could distinguish between healthy individuals and patients with hematological cancer [88].

#### Tumor educated platelets

Platelets are non-nucleated, small disc-shaped pieces of cell which are produced by megakaryocytes and found in the blood and spleen [89]. They aid in the formation of blood clots to slow/stop bleeding and allow wounds to heal [90, 91]. Blood platelets are unable to synthesize RNA on their own, and instead RNA is either endocytosed from circulation or derived from megakaryocytes.

Blood platelets can act as both local and systemic responders during cancer metastasis and tumorigenesis [92]. Tumor educated platelets (TEPs) are blood platelets which have been exposed to tumor induced platelet “education”. During this process, tumor cells can directly bind to the platelets “educating” them to contribute in tumor progression and metastasis [89], resulting in altered platelet behavior [92]. This change can be utilized as a biomarker to differentiate pan-cancer and non-small cell lung cancer (NSCLC) from healthy individuals [93, 94]. It has also been shown that TEPs can be used as a liquid biopsy for the detection of glioblastoma (GBM) [92] and sarcoma [95] cancer.

Advantages of TEPs in comparison to other blood-based biosources is related to their abundance, easy isolation, and ability to process RNA in response to external signals [91]. It has been shown that in most cancer patients the platelet RNA profiles are affected, independent of the type of tumor. However, the abundance of the tumor-associated RNAs varies between cancer patients [93].Best et al. demonstrated the ability to distinguish cancer patients from healthy individuals with a 96% accuracy in a cohort of 283 patients (228 with localized and metastasized cancer and 55 healthy individuals) using mRNA sequencing of tumor-educated blood platelets [93]. Moreover, they were also able to differentiate between six different primary tumor types (non-small cell lung, colorectal, glioblastoma, pancreatic, hepatobiliary and breast cancer) with a 71% accuracy.

Despite the increasing interest over the past years towards the research of the diagnostic potential of TEPs, there is no evidence of a commercialized test on the market that employs them as detection marker. However, TEPs can be investigated through mRNA sequencing, thus making them a signaling marker accessible for investigation through commonly commercialized sequencing technologies.

#### Autoantibodies

Autoantibodies are a form of antibody which react with substances formed by a person’s own body (i.e., self-antigens) [96]. These self-antigens can be exclusive for a specific-cell type within one organ of the body or can be found in all cell-types, such as chromatin or centromeres [97]. Autoantibodies can be found in autoimmune diseases and cancer [98], and have been shown to be useful biomarkers of disease as well as give information relating to inflammation in patients with autoimmune disease [97].

Autoantibody testing has been shown to be successful in the earlier detection of lung cancer. Sullivan et al. and Healey et al. have investigated the potential of the Oncimmune’s EarlyCDT-Lung (Biodesix, USA) liquid biopsy in measuring serum autoantibodies to tumor-associated antigens; Haley et al. specifically looked at the application of autoantibodies for detection of indeterminate pulmonary nodules and obtained an area under the curve (AUC) value for the receiver operating characteristic curve of 0.743, with maximum sensitivity of 98% at a 49% specificity [99–101].

Research around tumor-associated autoantibodies is still a developing field and more understanding surrounding their complex molecular response against cancer antigens is required [102]. Oncimmune currently leads the market of autoantibodies investigation tests with their liquid biopsy technology, although other companies provide autoantibodies test, such as GeneCopoeia™ with their OmicsArray™ Antigen Microarrays [103].

#### Spectroscopic detection

An alternative liquid biopsy strategy employs vibrational spectroscopy, specifically attenuated total reflectance (ATR) Fourier transform infrared (FTIR), coupled with machine learning. The potential of FTIR spectroscopy to analyze biological specimens as a cancer diagnostic tool has been known for decades^80^. Biological specimens such as bile, blood, extracellular vesicles, and urine have been studied using FTIR spectroscopy to help find alternative cancer diagnosis methods, as well as cancer management techniques. FTIR is a simple, label—free, rapid, cheap, non-invasive, non-destructive analytical method [104]. Instruments are easy to operate, and a vast amount of biological information can be gained from minute volumes (µL) of biological fluids.

This is achieved through the precise identification of molecular conformations, functional groups, bonding types as well as intermolecular interactions [105]. In ATR-FTIR spectroscopy, the infrared (IR) light is directed through an internal reflection element (IRE) which has a high refractive index (e.g., diamond/silicon), and interacts with the sample. Spectroscopy is sensitive to both the tumor and non-tumor derived information and generates a pan-omic biological signature that represents the whole biochemical profile of the analysed sample, producing a snapshot of the whole tumor and immune response to cancer. Combined with complex data analysis systems, valuable diagnostic information about the health status of individual patients can be obtained, since the biochemical fingerprint variations and spectral band patterns are exclusive to the molecular alterations in a specific disease [104–106]. One main benefit of ATR-FTIR spectroscopy is that sample preparation is minimal, eliminating complex pre-analytical steps that can introduce variation into the dataset [107].

**Fig. 6:**
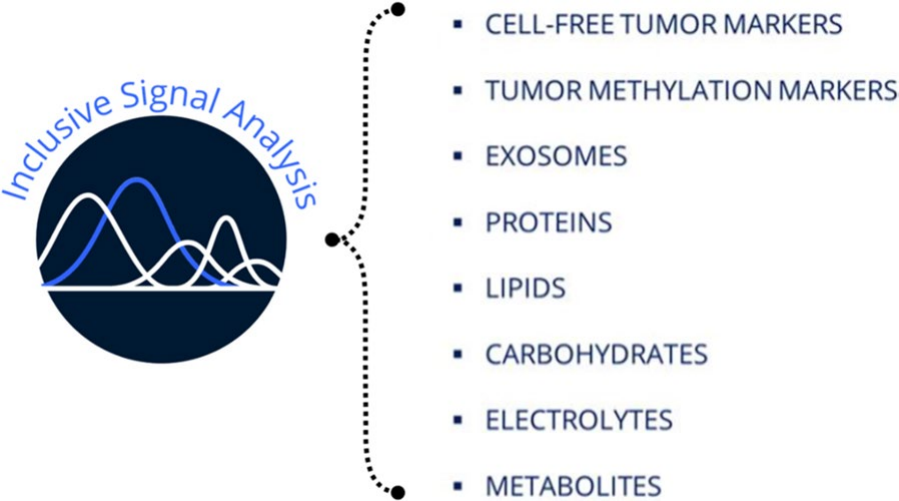
Liquid biopsy inclusive signal analysis

FTIR spectroscopy has been used for the interrogation of biofluids as a liquid biopsy tool for the detection of many cancers including; bladder [106], brain [108], ovarian [109], colorectal [110] and lung [111]. The spectroscopic analysis of blood and its derivatives (serum and plasma) addresses the intrinsic limitations of many genetic based liquid biopsies [41, 46, 108]. From this analysis a global signature is obtained, encompassing not only information surrounding the tumor but also on the body’s response to the tumor. This comes in the form of a complex biological absorbance spectrum containing a wealth of diagnostic information [41, 46, 108]. The ability to detect tumor and non-tumor derived information provides a snapshot of the overall response to cancer. The FTIR signal is inclusive; it embeds the analysis of metabolites, electrolytes, carbohydrates, lipids, proteins, exosomes, tumor methylation markers and cell-free tumor markers, as schematized in Fig. 6.

Drawbacks of spectroscopic detection in relation to cancer, include the inability of some techniques to provide information associated with the tumor in order to guide treatment [46]. In particular, when discerning the reason for the discrimination achieved spectral approaches are limited in their molecular resolution and can be difficult to pinpoint the exact biology responsible. Moreover, there is also a need for artificial intelligence in order to interpret the vast range of signals that spectroscopy obtains.

Cameron et al*.* conducted a prospective, analyst-blinded clinical study to demonstrate the utility of the spectroscopic brain cancer liquid biopsy [108]. Blood serum from 603 patients was collected and analyzed using the Dxcover® Brain Cancer liquid biopsy (Dxcover, UK). The recruited patients had either non–specific symptoms that could be indicative of a brain tumor or had been newly diagnosed with a brain tumor. The spectroscopic approach enabled algorithm tuning for greater sensitivity or specificity, which can be beneficial as the desired trade-off can differ between healthcare systems and diagnostic pathways. The sensitivity-tuned model gave a 96% sensitivity with 45% specificity, whereas when tuned for higher specificity, a sensitivity of 47% with 90% specificity was achieved. In addition, Theakstone et al*.* successfully managed to identify glioma cancer patients with tumor volumes as small as 0.2 cm^3^ via a spectroscopic liquid biopsy based on the absorbance of infrared radiation [112]. These findings highlight that spectroscopy can support the earlier diagnosis of brain cancer This is significant for the brain cancer community, as many other liquid biopsies are affected by the blood–brain barrier which inhibits the release of many biomarkers into the bloodstream. This blood test is sensitive to the body’s response to the tumor and non-tumor derived signals contributing to the machine learning classification.

### Cancer triage tests in the clinic

There are triage tests commercially available for certain cancers. For example, SelectMDx (MDx Health, Belgium) is a non-invasive urine liquid biopsy which measures the expression of two mRNA cancer-related biomarkers, and combines this information with clinical risk factors to stratify patients for clinically significant prostate cancer [113]. These results can help the physician determine if a patient can avoid a biopsy and return to routine screening, or if the patient may benefit from a biopsy for prostate cancer detection. From a validation cohort consisting of 715 patients with serum PSA less than 10 ng/mL, an AUC of 0.82 was achieved with a sensitivity of 89%, specificity of 53% and an NPV of 95%.

The ExoDx Prostate IntelliScore (EPI) (Exosome diagnostics, USA) is a non-invasive exosome-based liquid biopsy, which quantifies three RNA targets in urine exosomes [78]. The EPI test identifies patients at risk of high-grade prostate cancer. The test is carried out without the need for a digital rectal exam or a prostate massage and is independent of clinical variables. The main difference between the EPI assay and other assays such as SelectMDx which predict high-grade cancer, is the absence of clinical variables in the EPI algorithm. From the pooled analysis of three studies the combined cohort (n = 1212) gave an AUC of 0.70, with a sensitivity of 92.3%, specificity 30.1%, PPV 36.4% and a NPV of 90.1% [78]. A comparison of the sensitivity, specificity, turnaround time and costs of other single cancer liquid biopsy tests, including the ones mentioned above, can be seen in Table 1.

**Table 1:** Comparison of Liquid Biopsy Tests for Early Detection of Single Cancers

Company	Test	Target detection	Molecular origin	Specificity (%)	Sensitivity (%)	Turnaround time (cost/proposed cost)
Dxcover [108] (UK)	Dxcover brain cancer liquid biopsy*-*sensitivity tuned model	Brain cancer	Pan-omic Spectroscopic Assay from blood serum	45	96	1 day ($300)
	Dxcover brain cancer liquid biopsy-specificity tuned model			90	47	
Guardant health [114] (USA)	Shield^™^	Colorectal cancer	Shield is a qualitative blood test, intended to detect colorectal neoplasia through identifying genomic and epigenomic alterations in cfDNA, and proteomic changes in plasma from blood collected in guardant blood collection tubes	92.0	91.0	Around 2 weeks after the lab receives the samples [115] ($895) [116]
Novigenix [117] (Switzerland)	Colox	Colorectal cancer	Analysis of peripheral blood mononuclear cells isolated from blood sample	92.2	78.1	1–2 weeks. ($290) [118]
Biodesix [99] (USA)	Nodify cdt	Lung cancer	Autoantibodies on an enzyme-linked immunosorbent assay (ELISA) platform from blood	98	28	1 day ($649) [119]
Biodesix [120] (USA)	Nodify xl2	Lung cancer	Measures proteins from blood with liquid chromatography - mass spectrometry	44	97	4–5 days ($3850) [121]
Biodesix [100] (USA)	Oncimmune EarlyCDT-lung test stage I/II (Biodesix, USA)	Lung cancer	ELISA platform that measures autoantibodies from blood	90.3	52.2	~ 10 working days. ($84.98^a^/£70) [122]
	Oncimmune EarlyCDT-lung test stage III/IV (Biodesix, USA)			90.2	18.2	
Abcodia [123] (UK)	ROCA	Ovarian cancer	The ROCA test uses CA-125 measurements to establish a patient’s baseline levels of CA-125 to give an individualized profile of change over time, from a blood sample	87.6	87.1	Turnaround time - ($182.11^a^/£150) [124]
ExοDx [78] (USA)	ExoDx (EPI) prostate *Intelliscore*	Prostate cancer	Non-invasive exosome-based liquid biopsy, which quantifies three RNA targets in urine exosomes	30.1	92.3	1 week after lab receiving sample. ($795) [125]
MDxHealth [113] (Belgium)	SelectMdx	Prostate cancer	Measures the expression of two mRNA cancer-related biomarkers combined with clinical risk factors to stratify patients for clinically significant prostate cancer from a urine sample	53	89	5 business days. ($364.22^a^/£300) [126]
OPKO [127, 128] (USA)	4Kscore test	Prostate cancer	A follow-up blood test after an abnormal PSA or digital rectal exam which measures four prostate-specific kallikreins and clinical results to determine the probability of finding aggressive rostate cancer if a biopsy was performed	27.4	96.9	2–3 days from lab receiving sample. ($760) [129]

- represents data unable to source information on, ^a^represents that the currency was converted to dollars, using the exchange rate of $1.21 to £1

### Multi-cancer detection

Many cancers are not screened for on an individual basis as the prevalence rates in the general population are too low to make the process an effective intervention [130]. An alternative strategy is to screen for multiple cancers simultaneously in a single test. Detection of multiple cancers through a single analytical test would be transformative, specifically for people with less prevalent cancers that are currently not screened for [46]. Early signs of cancer can be non-specific and can easily be disregarded by both patients and practitioners since they are not indicative of a specific single organ for further testing. This can lead to a delay in testing and diagnosis for a patient, leading to a poorer prognosis. A rapid, low-cost test that can detect multiple cancer types could effectively provide an enhanced cohort of patients which have elevated ‘risk’ of cancer, to be prioritized for further diagnostic investigation [46].

Many technologies in the liquid biopsy field are targeting screening tests with high specificity to reduce the number of false positives. Klein et al*.* carried out a case-controlled observational study on 4077 patients to demonstrate the utility of a blood-based test. They used cfDNA sequencing and machine learning to detect cancer signals across vast cancer types and predict the cancer signal origin [11]. The objective of the study was to validate the blood test for use as a screening tool. The overall sensitivity achieved for cancer signal detection was 51.5% with a specificity of 99.5%. Blood-based tests are feasible, but early-stage detection remains a concern. Only 16.8% of stage I cancers were successfully identified, which is likely because of the lack of ctDNA released into the bloodstream in early-stage cancers.

The clinical utility of the Galleri test (Grail, USA) described by Klein et al. [11] is currently being validated through the NHS-Galleri trial, which aims to recruit 140,000 people ages 55 to 77 in the United Kingdom [131]. The study is currently enrolling patients by invitation, which have not been diagnosed or treated for cancer in the past three years [131]. The PATHFINDER study is a prospective, multi-center study which enrolled approximately 6,600 participants that will be followed for 12 months from the time of their enrollment [132]. The study aims to evaluate the implementation of an earlier version of the Galleri test in clinical practise. The test results will be communicated to health care providers and participants and used to help guide diagnostic workups [132].

PanSeer (Singlera Oncology, USA) is a blood-based screening test for the early detection of cancer [133]. The test is based on ctDNA methylation within plasma samples. In a retrospective, longitudinal study Singlera Oncology aimed to demonstrate the ability for the early detection of multiple cancer types up to four years prior to conventional diagnosis. They achieved an overall specificity of 96.1% and a sensitivity of 87.6% for post-diagnosis samples, 94.9% for pre-diagnosis samples [133]. Overall, the PanSeer liquid biopsy test was able to identify five types of cancer. This provides a preliminary demonstration of the ability of a blood test to detect multiple cancers types up to 4 years prior to conventional diagnosis utilizing methylation markers. Further work is still required to validate this methodology with prospective patient recruitment.

CancerSEEK (Exact Sciences, United States) is a liquid biopsy which combines assays for genetic alterations and protein biomarkers for the early detection of cancer [52]. CancerSEEK was used in a study of patients (n = 1005) that had been diagnosed with stage I-III cancers, examining eight cancer types (ovary, liver, stomach, pancreas, esophagus, colorectum, lung or breast). The test gave a specificity of > 99% with sensitivities of 43%, 73% and 78% for stages I, II and III respectively. These results are encouraging, but still over half of stage I cancers would be missed.

Cameron et al. analyzed the blood serum of 2094 patients in a large-scale multi-cancer study using the Dxcover^®^ Cancer Liquid Biopsy platform (Dxcover, UK) [46]. The aim of the study was to determine the ability of the platform to differentiate between non-cancer patients and various cancer types: brain, breast, colorectal, kidney, lung, ovarian, pancreatic, and prostate cancer. With a sensitivity-tuned model, focused on cancer versus asymptomatic non-cancer patients akin to screening an asymptomatic population, sensitivity was 98% and specificity of 58%. Alternatively, the specificity-tuned model had sensitivity of 56% and specificity of 99%. The key result for this study however lies in the ability to detect early-stage cancers (I and II) due to the analysis across biomolecular classes that originate from the tumor and from the immune response rather than just focusing on tumor related information. Cameron et al*.* demonstrate an ability to tune their approach to either highlight sensitivity or specificity with significant detection of early stage cancers via both methods [46]. These results demonstrate the potential of the Dxcover^®^ Cancer Liquid Biopsy as a rapid multi-cancer detection test for the identification of early-stage (I and II) cancers (Table 2).

**Table 2:** Sensitivity of Multi-cancer Signal Detection by Clinical Stage

Company	Test (cost/ proposed cost)	Molecular origin	Specificity (%)	Sensitivity			
				Stage I (%)	Stage II (%)	Stage III (%)	Stage IV (%)
Dxcover [46]^a^ (UK)	Dxcover cancer liquid biopsy ($300) (sensitivity tuned)	Spectroscopic pan-omics	58	99	97	99	98
Dxcover [46]^a^ (UK)	Dxcover cancer liquid biopsy ($300) (specificity tuned)	Spectroscopic pan-omics	99	64	51	62	57
Grail [11]^b^ (USA)	Galleri ($949) [134]	Methylomics	99.5	16.8	40.4	77.0	90.1
Thrive [52]^a^ (USA)	CancerSEEK ($-)	cfDNA and biomarkers	> 99	43	73	78	N/A

^a^Cancer versus asymptomatic Non—cancer patients

^b^Cancer versus Non-cancer (asymptomatic or symptomatic status unknown.) N.B. The different studies used different datasets, for a true comparison these technologies should be applied to the same dataset, - represents data unable to source information on

## Conclusion

**Fig. 7:**
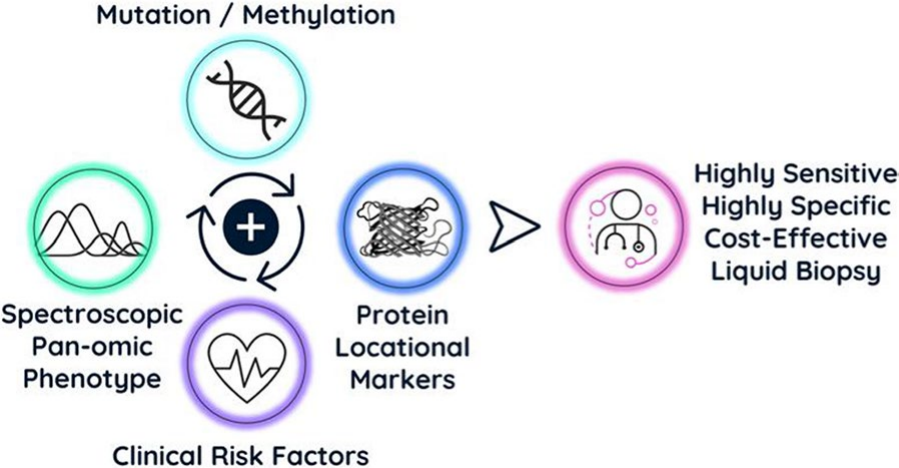
An effective test for multi-cancer detection can be achieved through a combination of various technologies

Liquid biopsies that can detect cancer early will improve patient prognosis and survival. The current definition of a liquid biopsy must be broadened to include both tumor and non-tumor derived information. The liquid biopsy market for early cancer detection is currently led by genetic testing of tumor derived biomarkers such as cfDNA. However, most current liquid biopsy techniques lack the detection capability required for early-stage cancers. There are though alternative methods that delve deeper into detecting non-tumor derived signals that dominate in early-stage cancers. A combination of a highly sensitive test with a highly specific orthogonal test (the combination of tests based upon fundamentally different phenomena) could be used on the enriched cohort as a second line test. [46]. This would provide an efficient system, capable of detecting early-stage tumors with both high sensitivity and high specificity [46] (Fig. 7).

## Data Availability

Not applicable.

## Abbreviations

ATR: Attenuated total reflectance
AUC: Area under the curve
CA-125: Carcinoma antigen- 125
CRUK: Cancer research UK
CT: Computed tomography
CTCs: Circulating tumor cells
cfDNA: Cell free DNA
cfRNA: Cell free RNA
ctDNA: Circulating tumor DNA
ctRNA: Circulating tumor ribonucleic acid
DNA: Deoxyribonucleic acid
ELISA: Enzyme linked immunosorbent assa
EPI: ExoDx prostate intelliscore
EVs: Extracellular vesicles
FDA: Food and drug administration
FTIR: Fourier transform infrared
GBM: Glioblastoma
HICs: High-income countries
IR: Infrared
IRE: Internal reflection element
LMICs: Low and middle-income countries
miRNA: Micro ribonucleic acid
MRI: Magnetic resonance imaging
mRNA: Messenger ribonucleic acid
MVs: Microvesicles
ncRNA: Non-coding ribonucleic acid
NSCLC: Non-small cell lung cancer
PSA: Prostate specific antigen
RNA: Ribonucleic acid
TEPs: Tumor educated platelets
